# BCL10 deficiency: Long-term immune reconstitution following hematopoietic stem cell transplantation

**DOI:** 10.70962/jhi.20260126

**Published:** 2026-10-20

**Authors:** Marwa Ali, Anas M. Alazami, Hamoud Al-Mousa

**Affiliations:** 1 Section of Allergy and Immunology, Department of Pediatrics, King Faisal Specialist Hospital & Research Center, Riyadh, Saudi Arabia; 2 Department of Translational Genomics, Genomic Medicine Centre of Excellence, King Faisal Specialist Hospital & Research Center, Riyadh, Saudi Arabia; 3 College of Medicine, Alfaisal University, Riyadh, Saudi Arabia

## Abstract

BCL10 deficiency is a rare combined immunodeficiency characterized by severe infections, chronic diarrhea, hypogammaglobulinemia, and impaired lymphocyte activation despite preserved lymphocyte counts. We report a novel homozygous *BCL10* variant with durable cure and immune reconstitution following hematopoietic stem cell transplantation.

## Teaser

Ali et al. describe a novel case of human BCL10 deficiency with a 13-year follow-up after hematopoietic stem cell transplantation. Their findings expand the clinical and genetic spectrum of this ultra-rare immunodeficiency and demonstrate that early transplantation can achieve durable immune reconstitution and long-term disease control.

Combined immunodeficiencies (CIDs) comprise a heterogeneous group of inborn errors of immunity characterized by impaired T cell function with variable effects on B cell and innate immune responses. Rapid advances in genomic technologies have reshaped the classification of CID by uncovering numerous disease-causing genetic variants and have considerably expanded our understanding of the molecular pathways required for normal immune function. Among these pathways, the caspase recruitment domain (CARD)–BCL10–MALT1 (CBM) signaling complex plays a central role in antigen receptor–mediated activation of nuclear factor κ-B (NF-κB). Following engagement of the T cell receptor or B cell receptor, assembly of the CBM complex triggers downstream activation of NF-κB, resulting in transcriptional programs essential for lymphocyte activation, proliferation, differentiation, cytokine production, and survival. Disruption of any component of this signaling axis can result in profound immune dysfunction and has led to the recognition of a growing group of disorders collectively termed “CBM-opathies.”

BCL10 is a critical adaptor protein within the CBM complex. Biallelic loss-of-function variants in *BCL10* result in severe CID (SCID) characterized by recurrent infections, chronic diarrhea, hypogammaglobulinemia, impaired lymphocyte activation, and failure to thrive. Since the first description of inherited BCL10 deficiency in 2014, only six affected individuals have been reported worldwide (1, 2, 3, 4, 5, 6). Among them, only one patient was described as successfully treated with hematopoietic stem cell transplantation (HSCT), with limited details regarding the transplant approach, conditioning regimen, engraftment, immune reconstitution, and long-term outcome (3). Consequently, the full clinical spectrum of BCL10 deficiency, its natural history, genotype–phenotype correlations, and optimal transplant strategies remain incompletely defined. Herein, we report a patient with a novel homozygous *BCL10* variant who presented with SCID and was successfully treated with HSCT. In addition, we review all reported patients to better define the clinical phenotype and therapeutic outcomes associated with this exceptionally rare disorder.

A Saudi female infant born to consanguineous parents was referred to our institution at 10 mo of age for evaluation of recurrent severe infections, chronic diarrhea, generalized eczema, and profound failure to thrive. She was born at term following an uncomplicated pregnancy and received routine childhood immunizations, including bacillus Calmette–Guérin (BCG) vaccination at birth. Her clinical manifestations began in early infancy with generalized cutaneous varicella infection requiring hospitalization and intravenous acyclovir therapy at 3 mo of age. Subsequently, she developed multiple serious infectious complications, including cellulitis at 6 mo and severe pneumonia at 7 mo requiring pediatric intensive care unit admission and ventilatory support; no causative bacterial pathogen was identified, and the diagnosis was based on clinical and radiological findings. Additional infections included cytomegalovirus (CMV) viremia at 8 mo, *Pseudomonas aeruginosa* otitis media at 10 mo, BCGitis with generalized lymphadenopathy at 12 mo, and mastoiditis at 13 mo. Chronic diarrhea had been present since 3 mo of age and resulted in severe malnutrition, hypoalbuminemia, and failure to thrive despite aggressive nutritional support. Physical examination demonstrated severe generalized eczema, hepatosplenomegaly, generalized lymphadenopathy, and anthropometric measurements below the third percentile for age. Family history was notable for two older brothers who died during infancy from overwhelming infections without a definitive diagnosis. This constellation of recurrent severe infections, chronic enteropathy, eczema, and growth failure with the family history prompted an extensive immunological evaluation for an underlying inborn error of immunity.

Initial immunological evaluation showed leukocytosis (18.5 × 10^9^/L) with marked absolute lymphocytosis (13.9 × 10^9^/L). Immunophenotyping demonstrated expanded T cell populations, with elevated absolute CD3^+^ (7,382 cells/μl), CD4^+^ (4,494 cells/μl), and CD8^+^ (2,277 cells/μl) counts, while CD19^+^ B cell and CD16^+^/CD56^+^ natural killer (NK) cell counts were 871 cells/μl and 293 cells/μl, respectively. Further phenotypic analysis demonstrated a reduced proportion of CD45RA^+^ T cells with a relative increase in CD45RO^+^ T cells. CD45RA^+^ cells accounted for 43% of CD3^+^ and 26% of CD4^+^ T cells, whereas CD45RO^+^ cells represented 38% and 36% of the respective populations. Despite the apparently preserved lymphocyte subset distribution, functional studies demonstrated profound T cell impairment. Lymphocyte proliferation assessed by ^3^H-thymidine incorporation showed markedly reduced responses to mitogenic stimulation, with proliferation to phytohemagglutinin, concanavalin A, and pooled allogeneic cells reaching only 25%, 9%, and 54% of normal control values, respectively. In contrast, the response to pokeweed mitogen was preserved at 125% of normal. These findings indicated severe functional T cell deficiency despite quantitatively normal circulating T cell numbers. Serum immunoglobulin levels were reduced for age, with IgG 2.4 g/L (age-adjusted reference range, 2.9–10.7 g/L), IgA <0.25 g/L (0.4–1.2 g/L), and IgM 0.18 g/L (0.3–1.5 g/L), consistent with pan-hypogammaglobulinemia. In view of the severe immune dysfunction and recurrent infections, the patient was started on monthly intravenous immunoglobulin (IVIG) replacement therapy, together with prophylaxis against *Pneumocystis jirovecii*, as well as antiviral and antifungal prophylactic agents.

At the time of her initial evaluation, ∼14 years before the current molecular diagnosis, her clinical presentation and immunological findings were highly suggestive of a leaky form of SCID. Given the genetic diagnostic capabilities available at that time, targeted Sanger sequencing of the most commonly implicated SCID-associated genes, including *RAG1*, *RAG2*, and *DCLRE1C*, was performed; however, no pathogenic variants were identified. Despite the absence of a molecular diagnosis, the severity of her clinical course strongly supported the diagnosis of a severe underlying combined inborn error of immunodeficiency. Consequently, HSCT was pursued as a potentially curative intervention. At 13 mo of age, she underwent a 6/6 HLA-matched unrelated cord blood transplantation (UCBT) following a reduced-intensity conditioning regimen comprising fludarabine (30 mg/m^2^/day, days −7 to −3), melphalan (140 mg/m^2^, day −2), and antithymocyte globulin (ATG; 5 mg/kg/day, days −5 to −2). The cord blood graft contained a total nucleated cell (TNC) dose of 0.09 × 10^9^/kg and a CD34^+^ cell dose of 0.05 × 10^6^/kg. Unfortunately, the transplant was complicated by primary graft failure with poor donor engraftment, resulting in persistent CMV viremia, recurrent infections, and ongoing gastrointestinal manifestations.

At 19 mo of age, a second 6/6 UCBT from a different donor was performed following myeloablative conditioning with intravenous busulfan administered every 6 h for 4 consecutive days (16 doses), on days −9 to −6 (1 mg/kg/dose), fludarabine 40 mg/m^2^/day for 4 days (−6 to −3), and ATG 2.5 mg/kg/day for 4 days (−5 to −2). The second cord blood graft contained a TNC dose of 0.04 × 10^9^/kg and a CD34^+^ cell dose of 0.03 × 10^6^/kg. Graft-versus-host disease prophylaxis consisted of cyclosporine and steroid. The early posttransplant period was complicated by grade I gastrointestinal graft-versus-host disease confirmed histologically and successfully treated with systemic high-dose corticosteroids and cyclosporine. Following the second UCBT, the patient demonstrated progressive immune reconstitution despite delayed establishment of detectable donor chimerism. Notably, at day +30, donor chimerism was 0% in both the T cell and myeloid compartments, accompanied by early posttransplant T cell lymphopenia, with absolute CD3^+^ and CD4^+^ counts of 303 and 130 cells/μl, respectively. By day +60, substantial lymphocyte recovery had occurred, with CD3^+^, CD4^+^, CD8^+^, and CD19^+^ counts increasing to 1,938, 1,157, 746, and 1,069 cells/μl, respectively. Despite this early numerical immune recovery, T cell donor chimerism remained undetectable at 0%, while myeloid donor chimerism had only become detectable at 6.8%. Donor contribution subsequently increased substantially. By day +180, T cell donor chimerism had risen to 83.35%, while myeloid donor chimerism reached 33.5%, accompanied by robust lymphocyte recovery. At 1 year, donor chimerism remained substantial, at 75% in T cells and 87% in the myeloid compartment. This progressive donor expansion was sustained during long-term follow-up, ultimately resulting in complete T cell donor chimerism (100%) and high-level myeloid donor chimerism (94%) at the most recent assessment. Long-term immune reconstitution was maintained, with absolute CD3^+^, CD4^+^, CD8^+^, CD19^+^, and CD56/16^+^ counts of 1,507, 800, 560, 302, and 463 cells/μl, respectively. Immunoglobulin replacement was successfully discontinued 15 mo after the second UCBT based on evidence of humoral immune reconstitution, including B cell recovery, endogenous immunoglobulin production, and the absence of recurrent or severe infections. Post-HSCT revaccination was initiated after discontinuation of IVIG, beginning with inactivated vaccines. Functional humoral recovery was subsequently demonstrated by protective vaccine-specific antibody responses to pneumococcal and tetanus vaccines, assessed ∼8 wk after immunization. Live attenuated vaccines were introduced at 36 mo after UCBT, following confirmation of adequate T cell immune reconstitution. She remains clinically well at long-term follow-up without recurrent severe or opportunistic infections, demonstrating sustained functional immune correction. The only notable late complication was the development of hyperthyroidism ∼6 years after HSCT, which has remained well controlled with medical therapy. Overall, her long-term outcome illustrates the potential for successful and durable immune restoration following HSCT, even in the setting of an initially complicated posttransplant course.

**Figure 1.**
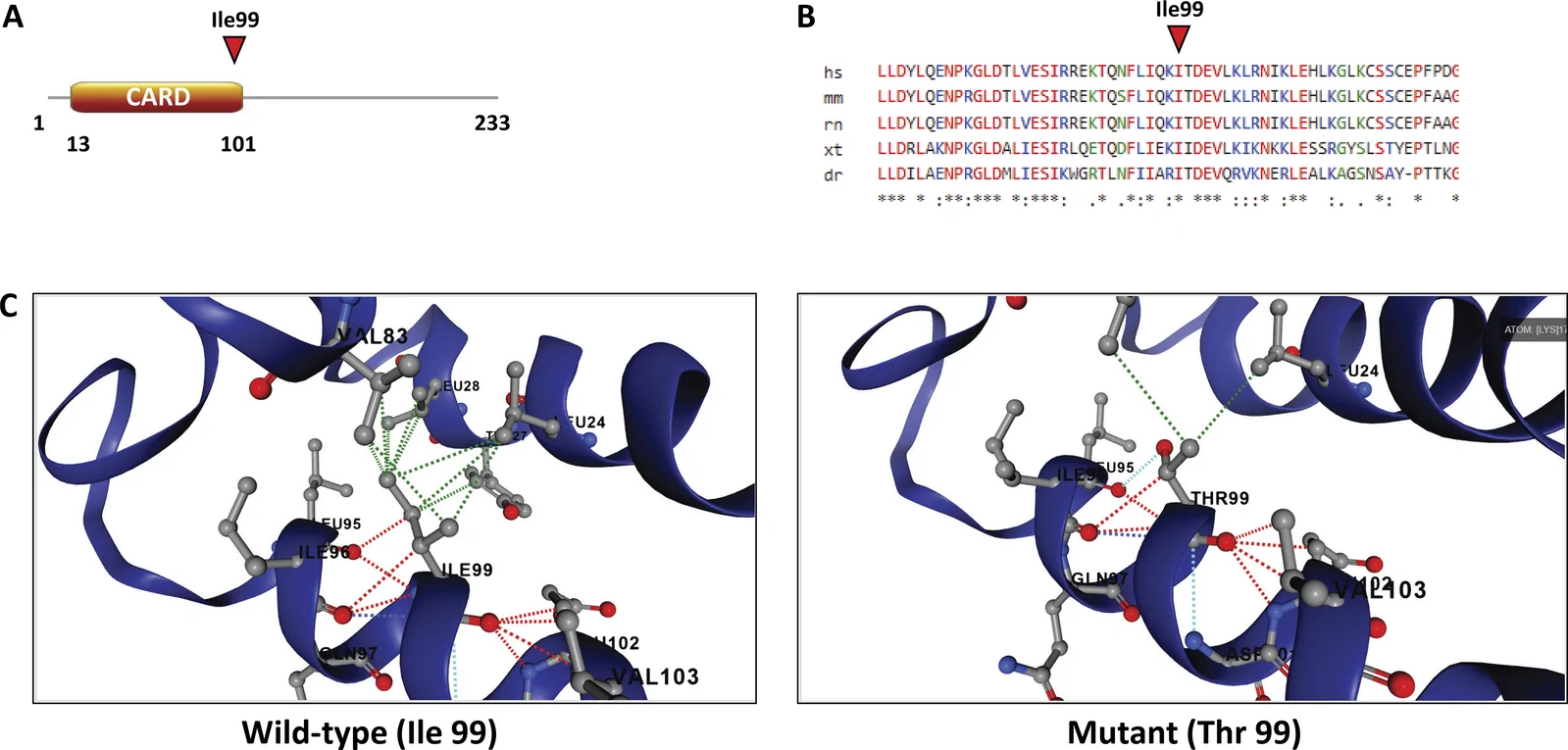
**Ile99 residue is highly conversed and is part of a hydrophobic network within the BCL10 core. (A)** Schematic of the human BCL10 protein. **(B)** Protein alignment shows the residue to be universally conserved across human (hs), mouse (mm), rat (rn), *Xenopus* (xt), and *Drosophila* (dr). **(C)** Wild-type residue is stabilized by an intricate network of hydrophobic interactions (indicated by multiple green lines) across Leu24, Tyr27, Leu28, and Val83. The polar Thr variant drastically compromises this microenvironment, reducing the hydrophobic interface to a solitary interaction vector (single green line) restricted to Leu24 and Val.

13 years after HSCT, advances in genomic diagnostics prompted re-evaluation of the underlying molecular etiology. Whole-exome sequencing performed on preserved pretransplant DNA identified a novel homozygous missense variant in *BCL10*, NM_003921.5: c.296T>C (p.Ile99Thr). This residue is located within the N-terminal CARD (Fig. 1 A), which spans most of the first 101 amino acids, and the region immediately upstream of Ile99 (residues 91–98) participates in BCL10 self-association, as well as interactions with external partners. BCL10 interacts with MALT1 directly via the fifth helix within the CARD, and mutations that disrupt proper CARD folding are known to severely destabilize the BCL10–MALT1 pairing. Protein alignment indicates that the amino acid is universally conserved across human, mouse, rat, *Xenopus*, and *Drosophila*, lending further weight to its predicted critical nature (Fig. 1 B). Based on its location and physicochemical properties, Ile99 is expected to contribute substantially to a dense-to-hydrophobic network within the CARD (Fig. 1 C). Substitution of isoleucine with threonine introduces a polar hydroxyl group into this hydrophobic environment. The DynaMut2 algorithm predicted a profound thermodynamic destabilization effect (ΔΔG = −3.27 kcal/mol) due to severe loss of hydrophobic packing density. This is therefore predicted to adversely affect CARD stability, with a potential consequent effect on BCL10–MALT1 interaction and downstream NF-κB signaling. Segregation analysis by Sanger sequencing confirmed heterozygosity in both parents, consistent with autosomal recessive inheritance. To our knowledge, this variant has not been previously reported in the literature or disease databases. Given that the molecular diagnosis was established more than a decade after transplantation, functional validation studies could not be performed. Nevertheless, the patient’s clinical phenotype was highly consistent with the growing spectrum of immune manifestations associated with human BCL10 deficiency. The identification of this novel variant provided a unifying molecular explanation for her disease and enabled definitive genetic counseling for the family.

Including the present patient, only seven individuals with genetically confirmed BCL10 deficiency have been reported worldwide (1, 2, 3, 4, 5, 6). Most were born to consanguineous families and presented during early infancy with severe and recurrent infections, reflecting the profound immune dysfunction associated with this disorder. Cases have been reported from diverse geographic regions, including North America, India, Oman, Saudi Arabia, and Europe, highlighting the global yet exceedingly rare occurrence of this condition. The clinical, immunological, genetic, and transplant characteristics of all published patients, together with the present case, are summarized in Table 1. Despite the limited number of reported patients, the clinical phenotype of BCL10 deficiency appears remarkably consistent. Disease onset typically occurs within the first months of life and is characterized by recurrent and often severe respiratory tract infections. Chronic diarrhea and failure to thrive were observed in most patients. Severe viral infections were a particularly prominent feature and included adenovirus, CMV, respiratory syncytial virus, influenza, varicella-zoster virus, and other respiratory viral pathogens. Opportunistic infections were also common and encompassed *P. jirovecii* pneumonia, disseminated adenoviral disease, invasive fungal infections, and complications related to BCG vaccination. Notably, several reported patients died from overwhelming viral or opportunistic infections before a definitive diagnosis could be established or curative treatment could be undertaken, underscoring the aggressive natural history and high mortality associated with untreated BCL10 deficiency.

**Table 1.** Genetic, clinical, and immunological characteristics of all reported patients with BCL10 deficiency

​	Nationality/sex	Clinical phenotype	Laboratory findings	Outcome	Genotype		
					Zygosity	Nucleotide level (NM_003921.5)	Protein level
P1 (1)	Amerindian male	• Consanguineous parents • 6 mo: gastroenteritis, respiratory infections • 8 mo: viral infections (flu A and B, adenovirus, RSV); acute secondary respiratory failure, oral candidiasis, and diaper dermatitis • 13 mo: prolonged diarrhea • 18 mo: diarrhea/active chronic colitis • 2 years 5 mo: acute gastroenteritis (adenovirus) and respiratory infection (RSV) • 2 years 8 mo: diarrhea (adenovirus); chronic nonspecific colitis • 2 years 10 mo: seizures and status epilepticus; secondary diffuse leukoencephalopathy • 3 years 4 mo: diarrhea (*Clostridioides difficile*); suspected encephalitis of unknown etiology • Died due to respiratory failure	• Normal total numbers of T and B cells • Profound deficit of memory T and B cells • Absent Treg • The T cells responded to PHA, ConA, and pokeweed, but not to anti-CD3/CD28 • Low level of all immunoglobulin subsets	Deceased	Homozygous	c.57+1G>A	Loss of expression
P2 (2)	Indian male	• Consanguineous parents • Had a female sibling with hypogammaglobulinemia; died from fungal pneumonia • 1 mo: BCGitis • 6 mo: severe viral LRTI and palatal ulcers • 8 and 10 mo: LRTI not requiring hospitalization • 11 mo: acute onset respiratory distress	• Normal total T cell number • Deficit memory T cells and B cells • Low level of all immunoglobulin subsets	No details reported	Homozygous	c.262C>T	p.R88X Loss of expression
P3 (3)	Saudi female	• Consanguineous parents • Had a female sibling who died at 15 mo of age due to BCGitis and bacterial sepsis • History of oral thrush • 12 mo: bacterial pneumonia that required hospitalization	• Normal total T cells • Deficit memory T and B cells • Reduced Tregs and TFH • Normal IgG; low IgA and IgM	Received HSCT from matched sibling donor	Homozygous	c.187A>T	p.K63X Loss of expression
P4 (4)	Omani female	• 40 days: scalp dermatitis progressing to involve the trunk and axillae • 4 mo: lower respiratory tract infection • 4–7 mo: admitted recurrently for respiratory infections • Adenoviral viremia and enteritis • PCP pneumonitis • Three episodes of ear infection • History of chronic diarrhea	• Normal total T cells • Low total B cells • Absent vaccine response • Absent proliferation in PHA • Low level of all immunoglobulin subsets	Deceased	Homozygous	c.271A>C	p.Thr91Pro Protein expression not done
P5 (5)	Saudi male	• Consanguineous parents • Patient presented at 2 mo of age: developmental delay, severe eczema, respiratory culture positive for *Moraxella catarrhalis*, pneumonia due to *Klebsiella pneumoniae*, skin infection due to MRSA, molluscum contagiosum, and *Microsporum canis*	• Normal total T cells and slight low B and NK cells • Low level IgG and IgM	On prophylactic measures	Homozygous	c.125G>A	p.R42H Low level of protein expression
P6 (6)	Balkan male	• Nonconsanguineous parents • 3 mo: started chronic diarrhea • 5 mo: severe adenovirus pneumonitis requiring invasive ventilation; progressed to adenovirus viremia and adenovirus gastroenteritis • PCP infection • Nutritional status—failure to thrive	• Nearly complete absence of memory T cells • Low IgG; IgM, IgA just above detection level • Absent lymphocyte proliferation following PHA and anti-CD3/anti-CD28	Deceased	Homozygous	c.57+1_57+4del	Loss of expression
P7^a^	Saudi female	• Consanguineous parents • History of early deaths of two siblings (boys; passed secondary to sepsis/infections) • Multiple early deaths of unknown cause in the family • History of chronic diarrhea since age of 3 mo • Had generalized severe atopic dermatitis • 3 mo: severe varicella infection • 6 mo: cellulitis • 7 mo: acute URTI (tonsillitis), which progressed to secondary bacterial pneumonia that require her escalation to HFOV in PICU • 8 mo: CMV viremia • 10 mo: secretory otitis media positive for *P. aeruginosa* • 12 mo: BCGitis followed by fungal sepsis • Nutritional status—failure to thrive	• High numbers of CD4^+^ and CD8^+^ T cells • Reduced levels of B and NK cells • Severe depression in lymphocyte response to PHA, ConA • Moderate depression in lymphocyte response to pooled allogeneic cells • Normal response to pokeweed • Low level of all immunoglobulin subsets	Received HSCT from matched unrelated donor; cord transplant	Homozygous	c.296T>C	p.Ile99Thr Protein expression not done

PHA,  phytohemagglutinin; ConA, concanavalin A; IVIG, intravenous immunoglobulins, LRTI, lower respiratory tract infection; Treg, regulatory T cells; TFH, T follicular helper cells; PJP, pneumocystis jirovecii pneumonia; MRSA, methicillin-resistant Staphylococcus aureus; URTI, upper respiratory tract infection; HFOV, high-frequency oscillatory ventilation; PICU, pediatric intensive care unit.

a Reported new patient.

Owing to the extreme rarity of *BCL10* deficiency, genotype–phenotype correlations remain incompletely understood. The pathogenic variants reported to date encompass a broad spectrum of molecular defects, including nonsense, splice-site, missense, and deletion variants. Despite this genetic heterogeneity, the clinical and immunological phenotype appears strikingly uniform across affected individuals. This relative phenotypic consistency suggests that diverse pathogenic variants ultimately converge on a common mechanism of impaired CBM complex signaling and NF-κB activation. Immunologically, BCL10 deficiency is characterized by a distinctive phenotype in which quantitative lymphocyte abnormalities may be relatively modest despite profound functional immune impairment. Absolute T cell numbers are frequently preserved or only mildly reduced, potentially obscuring the severity of the underlying defect if assessment is limited to lymphocyte enumeration alone. In contrast, functional studies consistently demonstrate severe impairment of T cell activation and proliferation in response to mitogenic stimulation. Most reported patients have also exhibited marked reductions in memory T and B cell subsets, impaired antigen-specific immune responses, and pan-hypogammaglobulinemia requiring immunoglobulin replacement therapy. These findings are consistent with the central role of BCL10 within the CBM signaling complex, which is essential for antigen receptor–mediated activation of NF-κB and the subsequent transcriptional programs required for lymphocyte activation, proliferation, differentiation, and survival. Accordingly, BCL10 deficiency primarily disrupts lymphocyte function and maturation rather than lymphocyte development itself. As a result, affected individuals may have apparently normal numbers of circulating T and B lymphocytes while remaining profoundly immunocompromised and highly susceptible to severe, recurrent, and opportunistic infections. This clinicopathologic dissociation underscores the importance of comprehensive functional immune testing in patients with suspected CID, particularly when conventional lymphocyte subset analysis appears relatively preserved.

An important consequence of this phenotype is the potential failure of newborn screening to identify affected patients. T cell receptor excision circle–based newborn screening primarily detects disorders associated with reduced thymic output and T cell lymphopenia. Because patients with BCL10 deficiency typically maintain normal numbers of naïve T cells, newborn screening results may remain normal despite profound functional immune impairment. Indeed, recent reports have documented patients with BCL10 deficiency who escaped newborn screening (6). Therefore, clinicians should maintain a high index of suspicion in infants presenting with chronic diarrhea, recurrent infections, failure to thrive, and hypogammaglobulinemia despite apparently normal lymphocyte counts.

BCL10 deficiency also contributes to the expanding spectrum of human CBM-opathies. Although defects in CARD11, CARD9, MALT1, and BCL10 affect a shared signaling pathway, significant phenotypic heterogeneity exists among these disorders. CARD9 deficiency predominantly affects antifungal immunity, whereas CARD11 and MALT1 deficiencies may present with CID, atopy, immune dysregulation, or inflammatory disease. In contrast, BCL10 deficiency appears to produce a relatively homogeneous phenotype characterized predominantly by SCID and susceptibility to life-threatening infections.

The present report also contributes valuable long-term transplant outcome data. Because the primary defect resides within hematopoietic cells, HSCT represents the most logical curative strategy. Nevertheless, published transplant experience remains extremely limited owing to the rarity of the disorder, and several reported patients died before transplantation could be performed, highlighting the aggressive natural history of BCL10 deficiency and the importance of early diagnosis and timely consideration of HSCT. Importantly, the only previously reported patient with BCL10 deficiency who underwent HSCT was also treated at our center. He underwent transplantation at 15 mo of age from an HLA-matched sibling donor following myeloablative conditioning with busulfan, fludarabine, and ATG. We now provide updated long-term follow-up showing that at 6 years after HSCT, he remains clinically well with complete donor chimerism (100%) in both lymphoid and myeloid compartments, sustained immune reconstitution, independence from immunoglobulin replacement, and protective antibody responses following revaccination. The present patient further extends the transplant experience in BCL10 deficiency, demonstrating durable immune correction more than a decade after UCBT despite delayed establishment of donor chimerism. Interestingly, the absence of detectable donor chimerism during the early posttransplant period did not predict subsequent graft failure. Rather, the progressive increase in donor contribution over time suggests delayed establishment and expansion of donor hematopoiesis. This may have been influenced by the relatively low cell dose of the cord blood graft. In addition, given the profound functional lymphocyte impairment characteristic of BCL10 deficiency, donor-derived lymphocytes may have acquired a selective functional advantage over residual recipient cells, potentially contributing to the progressive predominance of donor T cells. This interpretation remains speculative. Nevertheless, the longitudinal course demonstrates that undetectable donor chimerism early after UCBT did not preclude subsequent durable donor engraftment, complete T cell donor chimerism, and sustained immune reconstitution. Together, these two long-term outcomes demonstrate that successful HSCT can achieve sustained donor engraftment and durable restoration of immune function in BCL10 deficiency and provide further support for HSCT as a definitive therapeutic option for this severe disorder.

## Ethics statement

### Informed consent

Written informed consent for publication was obtained from the patient’s legal guardians.

### Ethical approval

This study was approved by the Institutional Review Board of King Faisal Specialist Hospital & Research Center.

## Data Availability

The data supporting the findings of this study are available from the corresponding author upon reasonable request, subject to institutional and ethical restrictions.
